# Forestalling Hungry Bone Syndrome after Parathyroidectomy in Patients with Primary and Renal Hyperparathyroidism

**DOI:** 10.3390/diagnostics13111953

**Published:** 2023-06-02

**Authors:** Mara Carsote, Claudiu Nistor

**Affiliations:** 1 Department of Endocrinology, Carol Davila University of Medicine and Pharmacy & C.I. Parhon National Institute of Endocrinology, Aviatorilor Ave. 34-38, Sector 1, 011863 Bucharest, Romania; 2 Department 4—Cardio-Thoracic Pathology, Thoracic Surgery II Discipline, Carol Davila University of Medicine and Pharmacy & Thoracic Surgery Department, Dr. Carol Davila Central Emergency University Military Hospital, 050474 Bucharest, Romania; ncd58@yahoo.com

**Keywords:** hungry bone syndrome, parathyroidectomy, surgery, calcium, parathormone, hyperparathyroidism, chronic kidney disease, renal hyperparathyroidism, diagnostic, parathyroid adenoma

## Abstract

Hungry bone syndrome (HBS), severe hypocalcemia following parathyroidectomy (PTX) due to rapid drop of PTH (parathormone) after a previous long term elevated concentration in primary (PHPT) or renal hyperparathyroidism (RHPT), impairs the outcome of underlying parathyroid disease. Objective: overview HBS following PTx according to a dual perspective: pre- and post-operative outcome in PHPT and RHPT. This is a case- and study-based narrative review. Inclusion criteria: key research words “hungry bone syndrome” and “parathyroidectomy”; PubMed access; in extenso articles; publication timeline from Inception to April 2023. Exclusion criteria: non-PTx-related HBS; hypoparathyroidism following PTx. We identified 120 original studies covering different levels of statistical evidence. We are not aware of a larger analysis on published cases concerning HBS (N = 14,349). PHPT: 14 studies (N = 1545 patients, maximum 425 participants per study), and 36 case reports (N = 37), a total of 1582 adults, aged between 20 and 72. Pediatric PHPT: 3 studies (N = 232, maximum of 182 participants per study), and 15 case reports (N = 19), a total of 251 patients, aged between 6 and 18. RHPT: 27 studies (N = 12,468 individuals, the largest cohort of 7171) and 25 case reports/series (N = 48), a total of 12,516 persons, aged between 23 and 74. HBS involves an early post-operatory (emergency) phase (EP) followed by a recovery phase (RP). EP is due to severe hypocalcemia with various clinical elements (<8.4 mg/dL) with non-low PTH (to be differentiated from hypoparathyroidism), starting with day 3 (1 to 7) with a 3-day duration (up to 30) requiring prompt intravenous calcium (Ca) intervention and vitamin D (VD) (mostly calcitriol) replacement. Hypophosphatemia and hypomagnesiemia may be found. RP: mildly/asymptomatic hypocalcemia controlled under oral Ca+VD for maximum 12 months (protracted HBS is up to 42 months). RHPT associates a higher risk of developing HBS as compared to PHPT. HBS prevalence varied from 15% to 25% up to 75–92% in RHPT, while in PHPT, mostly one out of five adults, respectively, one out of three children and teenagers might be affected (if any, depending on study). In PHPT, there were four clusters of HBS indicators. The first (mostly important) is represented by pre-operatory biochemistry and hormonal panel, especially, increased PTH and alkaline phosphatase (additional indicators were elevated blood urea nitrogen, and a high serum calcium). The second category is the clinical presentation: an older age for adults (yet, not all authors agree); particular skeleton involvement (level of case reports) such as brown tumors and osteitis fibrosa cystica; insufficient evidence for the patients with osteoporosis or those admitted for a parathyroid crisis. The third category involves parathyroid tumor features (increased weight and diameter; giant, atypical, carcinomas, some ectopic adenomas). The fourth category relates to the intra-operatory and early post-surgery management, meaning an associated thyroid surgery and, maybe, a prolonged PTx time (but this is still an open issue) increases the risk, as opposite to prompt recognition of HBS based on calcium (and PTH) assays and rapid intervention (specific interventional protocols are rather used in RHPT than in PHPT). Two important aspects are not clarified yet: the use of pre-operatory bisphosphonates and the role of 25-hydroxyitamin D assay as pointer of HBS. In RHPT, we mentioned three types of evidence. Firstly, risk factors for HBS with a solid level of statistical evidence: younger age at PTx, pre-operatory elevated bone alkaline phosphatase, and PTH, respectively, normal/low serum calcium. The second group includes active interventional (hospital-based) protocols that either reduce the rate or improve the severity of HBS, in addition to an adequate use of dialysis following PTx. The third category involves data with inconsistent evidence that might be the objective of future studies to a better understanding; for instance, longer pre-surgery dialysis duration, obesity, an elevated pre-operatory calcitonin, prior use of cinalcet, the co-presence of brown tumors, and osteitis fibrosa cystica as seen in PHPT. HBS remains a rare complication following PTx, yet extremely severe and with a certain level of predictability; thus, the importance of being adequately identified and managed. The pre-operatory spectrum of assessments is based on biochemistry and hormonal panel in addition to a specific (mostly severe) clinical presentation while the parathyroid tumor itself might provide useful insights as potential risk factors. Particularly in RHPT, prompt interventional protocols of electrolytes surveillance and replacement, despite not being yet a matter of a unified, HBS-specific guideline, prevent symptomatic hypocalcemia, reduce the hospitalization stay, and the re-admission rates.

## 1. Introduction

Hungry bone syndrome (HBS), rarely named “bone starvation syndrome”, severe hypocalcemia following parathyroidectomy (PTX) due to rapid drop of PTH after a previous long term elevated concentration and associated bone remineralization, impairs the outcome of underlying parathyroid (PT) disease by affecting the quality of life, prolonged hospitalization stay, and increased post-operatory re-admission rate [1,2,3].

HBS prevalence widely varies according to studied population, 15–25% to 92% of patients diagnosed with renal hyperparathyroidism (RHPT), in cases with primary hyperparathyroidism (PHPT), might not be identified at all, but overall prevalence accounts for up to 15–20% of individuals with PHPT; generally, a more important risk of developing HBS is registered in participants with impaired renal function [2,3,4,5,6].

Hypocalcemia (usually below the value of 8.2–8.4 mg/dL), a typically severe and even life-threatening unless prompt intervention, arises within the third (varying between the first/second and the fourth–seventh) post-operatory day and it usually progresses through a 3-day period of time up to 30 days, requiring intravenous calcium replacement. Associated hypophosphatemia, hypomagnesiemia, and, exceptionally, hyperkaliemia (in patients undergoing chronic dialysis) are identified [3,7,8,9]. This severe, but transitory, event is followed by mild or asymptomatic hypocalcemia requiring oral calcium and vitamin D substitution, particularly calcitriol, which may take a few months up to a year in order to register the restauration of normal mineral metabolism without the help of any medication [3,10,11]. Early after PT surgery, normal or high (but lower than pre-operatory level) parathyroid hormone (PTH) is essential for establishing HBS diagnostic since non-low PTH is the clue to differentiate the condition from post-surgery hypoparathyroidism (low PTH) [12,13].

Pre-operatory assessments that might be a clue for further developing HBS after PTx vary; the most common are extremely high serum PTH and bone formation marker alkaline phosphatase (AP), noting that HBS involves an increased osteoblastic activity in association with a normal or low osteoclastic activity. Correction of hypercalcemia and starting calcium replacement from the first day of PTx might improve the outcome of HBS [7,14,15].

The pre-operatory use of anti-resorptives such as bisphosphonates (for example, pamidronate or zoledronic acid) in PHPT is controversial to associate benefits for HBS, but some authors reported it [16,17,18,19]. Moreover, PT and bone imaging assessments before surgery in addition to histological information through bone biopsy for associated pathological skeletal masses and through post-PTx examination of PTs might suggest other contributors to HBS such as: giant PT or atypical tumors (typically larger than 2 cm diameter), PT carcinoma, respectively, the presence of brown skeletal tumors, and osteitis fibrosis cystica [7,14,20,21].

In patients with chronic renal failure, maintaining an abnormal calcium phosphate product might help the evolution of HBS, as well as other outcomes belonging to the cardiovascular and hematologic profile [22]. RHPT (also called secondary HPT, but it should be differentiated from vitamin D deficiency-associated secondary HPT which is not a matter of PT surgery, but of adequate oral vitamin D replacement) is a complication of chronic kidney disease usually starting with the third stage. The use of calcimimetics as cinalcet might help, but failure of overall medical intervention, for instance, a PTH above 800 pg/mL in association with high serum calcium, hyperphosphatemia, pruritus, or bone pain represents an indication of PTx [2,23,24,25,26].

Over the decades, HBS was listed among the severe side of post-PTx complications, a heterogeneous picture that includes vocal cord palsy, local hemorrhage, transitory or permanent hypocalcemia/hypoparathyroidism, or even peri-operatory mortality [27,28,29,30,31,32,33].

### Aim

Our objective was to overview HBS following PTx according to a dual perspective: the status before surgery as a potential clue for developing HBS and post-operative outcome in individuals with normal renal function diagnosed with PHPT or in those associating chronic kidney disease that induced RHPT.

## 2. Methods

This was a case- and study-based narrative review. We revised papers according to the following inclusion criteria: key research words “hungry bone syndrome” in combination with “parathyroidectomy”; PubMed-access; in extenso articles; publication timeline from Inception to April 2023, and, respectively, exclusion criteria: non-PTx-related HBS; hypoparathyroidism following PTx; types of papers other than original studies (but we took case reports and case series into consideration as well).

**Figure 1:**
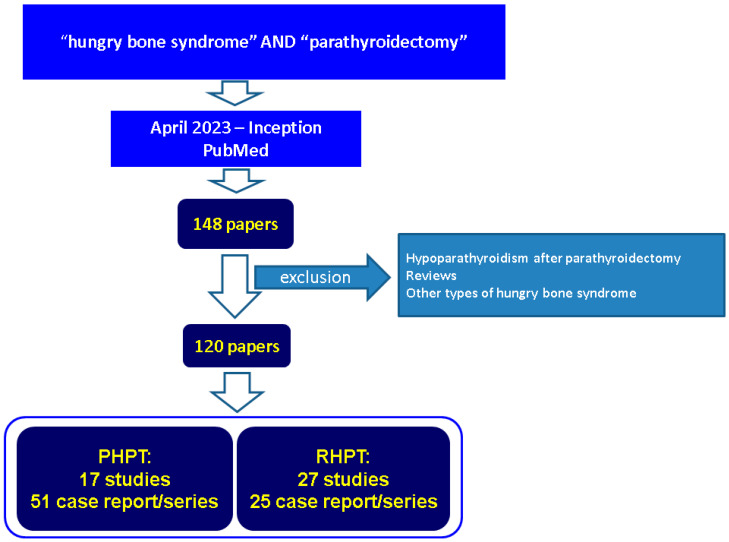
Flowchart diagram of research according to our methods. Abbreviations: PHPT = primary hyperparathyroidism; RHPT = renal hyperparathyroidism.

According to our methodology we identified 148 papers starting with 1976 concerning “HBS” and “PTx” in title and/or abstract (a total of 250 papers are identified only using the search term “HBS”). We manually searched each of them, excluded the duplicates, and selected 120 original studies covering different levels of statistical evidence for the final analysis (Figure 1).

## 3. PTx-Related HBS

We included in the final analysis (n = 120 original studies): 17 original studies in adult and pediatric population diagnosed with PHPT (from 2022 to 1988) and 27 studies in adult participants confirmed with RHPT (from 2023 to 2000, we did not find any particularly cohort addressing pediatric individuals with RHPT), respectively, 76 case reports and series (a maximum of 10 patients per article) including adults, children, and teenagers confirmed with PHPT and RHPT who developed post-PTx HBS (from 2023 to 1976).

### 3.1. Pre-Operatory Predictors of HBS in PHPT

HBS prevalence in adult population was heterogeneously reported and we looked at any type of original study that specifically reported HBS as a consequence of PTx (N = 14 studies with adult population, the most recent was from 2022, while the oldest was from 1988) [6,34,35,36,37,38,39,40,41,42,43,44,45,46] (Table 1).

The lowest rate of HBS was 2.4%; the highest was of 28.7%, while most data were concentrated between the values of 12% and 25%; all these studies were retrospective with 2 exceptions (one case-controlled and one interventional study) [6,34,35,36,37,38,39,40,41,42,43,44,45,46]. The rate of 2.4% was confirmed in a two-centered, retrospective study on 164 participants from Singapore diagnosed with PHPT (between 2012 and 2019). HBS was defined based on the followings criteria: a value of albumin-adjusted calcium below or at highest 2.1 mmol/L in association with normal/high iPTH starting within the third day after PTx with a duration of at least 3 days and it was found to be positively correlated with a longer hospitalization (20 days vs. 2 days in patients without HBS, *p* < 0.001). Pre-operatory predictors of HBS were not found to be age, sex distribution, use of anti-resorptive drugs, vitamin D deficiency, but the values of iPTH and AP (for every 10 units increase of these pre-operatory parameters, the risk of developing post-surgery HBS was statistically significant higher by 14%, respective 11%) [35].

Tang et al. [34] performed a multi-center, retrospective study in 28 adults (mean age of 56 years, aged between 23 and 83 years, 68% of them were females) diagnosed with atypical PT adenomas-associated PHPT (between 2000 and 2018) complicated with renal function damage and stones in one third of them, and bone mineral density loss in one fifths of them. Post-operatory rate of HBS was of 7%, while recurrent laryngeal nerve paresis or paralysis was of 14% [34]. HBS prevalence of 19.3% following PTx (42% of the participants had a minimally invasive procedure) for PHPT (N = 62, mean age of 47 years, 88% female predominance) was identified in a single-center, retrospective study from Algeria (post-operatory hypocalcemia of any degree was reported in 59.7% of the participants, while severe hypocalcemia was noted in 41.9% of the entire cohort) [36]. The issue of atypical PT adenomas (recently renamed “atypical PT tumors” according to WHO 2022 classification) [47] as associating a higher risk of post-surgery HBS was raised by another case initially admitted for a parathyroid crisis. This was a 56-year-old male (also associating anemia) who developed a 6-month HBS after en bloc resection. The authors reported a similar case of atypical PT adenoma on a 64-year-old female presented for a PT crisis and an acute form of a chronic renal damage as onset of PHPT; she underwent a selective PTx with persistent HPT and no HBS, suggesting that persistent HPT might be the opposite scenario for the patients who develop HBS [48]. Generally, a PT crisis is an extremely severe and rare complication of hormonal anomalies in PHPT. This severe clinical complication is still an open issue if it represents an independent risk factor for HBS [48,49].

Giant PT adenomas (of more than 2–3 g) display a higher rate of bone complications in terms of brown tumors and osteitis fibrosa cystica which themselves are prone to post-PTX HBS, noting that usually the size of PT tumor associates with the levels of serum PTH [50]. Alvarez-Payares et al. [7] added a new such case, a 55-year-old female with a giant PT adenoma (of 5 cm, and a weight of 16 g) who was post-operatory re-admitted for HBS (the symptoms started 72 h after surgery and the patient remained hospitalized for one month, also associating an aggravation of chondrocalcinosis and an acute episode of pulmonary embolism). The authors reviewed data on 2 medical databases concerning giant PT adenomas starting with 2009, since first report was introduced, until 2021 and found 24 studies; HBS rate was reported in 25% of the cases with RHPT (USA), and in 4–87% of the participants diagnosed with PHPT. HBS was found to be correlated with larger tumors (such as giant PT adenomas), a PTH value above 1000 pg/mL, serum AP concentration of 3 times above normal upper limit, an increased number of osteoclasts at bone biopsy, the co-presence of skeleton complications such as brown tumors, and osteititis fibrosa cystica [7]. A similar case of a giant PT adenoma (of 5.6 cm largest diameter, and a weight of 28.7 g) was admitted for high calcium (of 13.7 mg/dL) and PTH (of 1240 pg/mL); the 66-year-old female associated a 4-week episode of HBS starting from the first day after PTx [51]. Other particular histological profiles of PT tumors prone to HBS after their removal involves PT carcinoma, the level of statistical evidence remaining low due to the rarity of condition [35,52,53,54,55].

A study on 94 patients with PHPT showed that the group with HBS (representing 28.7% of the entire cohort) vs. those HBS-free had an associated (synchronously) thyroid surgery, a longer period of PTx, a higher PT tumor weight, and an increased iPTH level before surgery (pre-PTx hormone cutoff of 45 pg/mL correlated with a 90% risk in relationship to developing HBS) [39]. Notably, another study highlighted the same results concerning simultaneous thyroidectomy with PTx as increasing the risk of HBS. Kaya et al. [43] studied 62 participants with PHPT and 13.4% of them were confirmed with HBS; the post-operatory complication being correlated with pre-surgery AP, PTH, blood urea nitrogen (as a reflection of renal function), co-diagnosis of osteoporosis, and a pathologic report showing PT hyperplasia [43].

Brown tumors, located at ribs, clavicles, femur, pelvis, jaw (and even patella, according to the report of Irie et al. [56]), are skeletal cancer-like osseous masses underlying osteolysis associated with increased vascularization, being part of long standing PHPT and they seem to increase the risk of developing post-operatory HBS [56,57]. They may be multiple and a differentiation from a metastasis is required from the start, but, mostly, the biochemistry panel suggestive for a PT condition is enough not to perform a bone biopsy from the first detection in order to provide the surgical cure of PTH excess. Afterwards, the “wait and see” approach based on imagistic surveillance is useful since a spontaneous remission is expected if the underlying PT disease is remitted through PT tumor removal [58]. According to a systematic review from 2013, HBS seems more frequent in cases with PHPT complicated with PT bone disease vs. those without radiological evidence of bone involvement (25–90% vs. 0–6%) [59]. However, nowadays, the presentation of PHPT was reshaped to an early diagnostic of an asymptomatic or mildly symptomatic condition; thus, a less frequent skeleton involvement was reported, the opposite of previously reported cases complicated with osteitis fibrosa cystica and brown tumors [60,61].

The relationship with intensive vitamin D supplementation before PTx in order to prevent HBS was studied, noting that, generally, this type of pre-operatory medical intervention improves the component of hypovitaminosis D-associated secondary HPT and, potentially, the long term bone outcome regardless HBS [62,63]. One prospective, randomized, open study included 102 participants with PHPT who underwent PTx. Group 1 (N1 = 52) did not receive vitamin D supplements before surgery, the second group received cholecalciferol (1000–2000 IU per day or 5000 IU per week) in order to achieve a 25-hydroxyvitamin D level of >20 ng/mL (N2 = 25) or >30 ng/mL (N3 = 25). HBS prevalence was not statistically significant different (8%, 16%, and 23%) among the three mentioned subgroups, but, generally, HBS reached the statistical significance (*p* < 0.001) when correlated with a younger age at the moment PTx was performed (42.5 vs. 49.5 years), a higher AP at baseline as well as lower serum magnesium, lower 25-hydroxyvitamin D (11 vs. 17.3 ng/mL, but its pre-operatory level was not an independent predictor for HBS), and a higher pre-operatory bone mineral density (1.7 vs. 0.9 g/sqcm) when compare to HBS-free patients [37]. Kaderli et al. [40] did not establish a relationship between HBS after PTx for PHPT and 25-hydroxyvitamin levels assessed two days before surgery [40]. However, pre-surgery normalization or (at least) severe deficiency correction of 25-hydroxyvitamin D levels is mandatory outside the perspective of further developing HBS consecutive to PT removal [10]. For instance, according to one randomized, placebo-controlled, double-blind, single center study in 46 participants with PHPT (an average age of 58 years) showed that 2800 UI of choleciferol per day as active intervention for 26 weeks pre- and post-PTx was associated with an improvement before surgery in terms of decreased PTH by 17%, increased bone mineral density by 2.5% and lowered bone resorption marker CrossLaps by 22%. After PT operation, trabecular bone score improved while PTH remained lower in treated group [64].

Overall, the studies in adults with PHPT who reported HBS as a post-PTx complication (n = 14 studies, N = 1545 patients with PHPT) may be grouped depending on sample size in small sample (<50 patients per study)—6 studies (enrolling 19, 23, 28, 29, 45, respectively, 46 participants); medium size (between 50 and 100 individuals per study)—4 studies (of 62 persons—two of them, respectively, 82, and 92 participants); and large studies (more than 100 participants per study)—4 studies (cohorts of 102, 164, 385, and 425 patients). Additionally, we analyzed the data with a lower statistical significance, 36 case reports (no case series was identified in adults with PHPT and HBS) introducing 37 participants (one article included 2 cases) confirmed with HBS after PTx for PHPT. The timeline started from 1976, while the most recent paper was published in 2023. The youngest patient was of 20 years, the oldest of 72 years; a total number of 1582 adults were studied for HBS following surgery for PHPT [7,10,11,14,15,19,20,48,50,51,52,53,54,55,56,57,58,65,66,67,68,69,70,71,72,73,74,75,76,77,78,79,80,81,82,83] ((Table 2).

### 3.2. Pediatric PHPT and HBS

Most studies (in PHPT and RPHT) included patients of adult age at the moment of PTx [84,85]. Some authors suggested that the interpretation of age as a risk factor for HBS should be differentially regarded in PHPT (an older age is prone to HBS) vs. RHPT (a younger adult is at higher risk for post-PTx HBS), but this is not a homogenous observation. For example, older age was identified to contribute to HBS in 2 studies [38,46] or younger age in another [37] or age at surgery was not correlated to the HBS outcome in PHPT [35].

Whether pediatric PHPT represents a particular risk factor for HBS following PT removal is still an open issue; noting that generally PHPT is extremely rare in children and teenagers [86,87,88,89,90]. Our sample-based study identified a number of 232 participants (children and adolescents) enrolled in 3 retrospective studies (published in 2021, 2016, and 2010), two of small size (N = 35, between 1989 and 2019, respectively, N = 15, between 1993 and 2006), and another larger study (N = 182, between 2009 and 2012). Grossly, one third of the studied population developed post-PTX HBS which was more than found in adult population with PHPT [91,92,93] (Table 3).

A pediatric case series (N = 10 participants diagnosed with PHPT) identified a rate of 40% with respect to post-PTx HBS (N = 4) which seems higher than the above mentioned studies [94]. For example, one of these was a retrospective cohort on 35 participants confirmed with PHPT who were younger than 18 years (mean age of 15.2 years, 40% with minimally invasive PTx, cure rate of 97%, median post-PTx follow-up of 5 years); the authors identified HBS in 35% of the children and adolescents [91]. This prevalence was similar to the other pediatric study (N = 15 children and teenagers with mean age of 17 years) that reported a HBS prevalence of 33.3% [93].

Of note, in 2020, a case of a 14-year-old male was published regarding a PHPT-associated pelvic brown tumor (the first pediatric subject with such tumor at this site, according to Legault et al. [95]) and developed a 6-day episode of HBS following PTx (associating a calcium nadir of 1.98 mmol/L); the brown tumor remitted within one year [95]. In 2015, an 18-year-old female confirmed with an atypical PT adenoma (of 3.8 g) complicated with PHPT-related brown tumors (a pre-operatory peak PTH of 2551 pg/mL) was reported to develop a severe 3-month HBS followed by an extended late post-operatory phase to 42 months (in the meantime, still requiring oral calcium and vitamin D replacements), an exceptional condition described by Juárez-León et al. [96] as “protracted HBS” [96]. We identified a third pediatric case with brown tumors (published in 2020): a 13-year-old boy with PT carcinoma-related PHPT which complicated with post-operatory HBS; intravenous calcium regimes were mandatory also for a prolonged period of 3 weeks [88]. Similarly to adults, the presence of tumor-like skeleton involvement caused by massive PTH overproduction represented the hallmark of further HBS after the PT tumor removal [88,95,96]. A total of 10 adults presented brown tumors and/or osteitis fibrosa cystica in reported cases we could identify, and no study specifically addressed the issue of HBS to a larger scale concerning the forms with severe skeleton findings (most probably due to their rarity) [7,20,56,57,58,65,69,70,75,77].

Overall, the pediatric case-sample analysis revealed another 15 case reports/series, as follows: 1 patient per article (n = 11 reports), 2 participants per report (n = 2 articles), 5 cases per series (n = 1), and 10 cases per series (n = 1). A total of 30 participants of 18 years or younger were studied and reported in order to address the issue of HBS. The case series of 10 patients included 4 participants with HBS (a rate of 4/10), the one of 5 patients reported one case of HBS (a rate of 1/5), and another case series of 2 patients only identified one individual with HBS; thus, we conclude that 19 children and teenagers were diagnosed with HBS (on case report level of statistical evidence). In addition to the mentioned pediatric studies in PHPT which analyzed the issue of post-operatory HBS, there were a total of 251 young patients, the youngest being a 6-year-old [86,87,88,89,90,91,92,93,94,95,96,97,98,99,100,101,102,103] (Table 4).

### 3.3. Pre-Operatory HBS Predictors in Subjects with RPHT

The panel of pre-PTx parameters that might represent contributors to post-operatory HBS was studied in RHPT even more consistently than in PHPT (only with respect to adult population), taking into consideration that the general prevalence of HBS in RHPT is unanimously recognized as being more frequent than in PHPT.

As in PHPT, pre-surgery high iPTH and AP levels were identified as predictors of the starvation bone syndrome. For instance, we mention a single-center, retrospective study on 141 individuals with RHPT that identified a HBS prevalence of 32% (N1 = 46) following successful PTx with at least 3 parathyroid glands being auto-transplanted. Pre-operative predictors of HBS (univariate analysis) were: higher dialysis age, respectively, increased values of PTH, calcitonin, and AP (which was found to be an independent predictor of HBS; a cutoff of 199.5 U/L offering a sensitivity of 80.85%, and a specificity of 82.61% regarding post-operatory HBS prediction), while pre-surgery PTH was correlated with the duration of intravenous regime of calcium replacement and total calcium dose (via intravenous supplementation) amid HBS [104].

A retrospective study conducted by Ge et al. [105] in 115 participants with PTx for RHPT identified a much higher rate of 87.8% concerning post-operatory HBS; the syndrome occurrence was correlated with increased baseline AP and low serum calcium, while its severity was independently associated with high pre-surgery AP, iPTH, and, respectively, a younger age at the moment of surgery [105]. Similar results were shown by a retrospective study from 2020 that enrolled 130 participants with RHPT (between 2014 and 2020); 85.4% of them received hemodialysis, the others were under peritoneal dialysis; PT hyperplasia was confirmed in 90.8% of the cohort. A total of 82.3% of the individuals developed HBS (defined as symptomatic hypocalcemia or serum calcium below 8.4 mg/dL, either variant requiring intravenous calcium supplementation within first 72 h following PTx). The syndrome correlated with a younger age (of 45 years or younger), high AP levels (above 420 UI/L), pre-operatory iPTH (higher than 1000 pg/mL), as well as lack of pre-surgery hypercalcemia (meaning a serum calcium below 10.2 mg/dL) and, respectively, with a longer hospitalization duration (8 days in persons with HBS vs. 3 days for the patients without HBS, *p* < 0.01) [85]. With respect to the patients’ age, a small retrospective study (N = 37 participants with RHPT) showed that this parameter negatively correlated with the duration of post-PTx hospitalization and post-surgery hypocalcemia [106]. Another study on 84 patients with RHPT identified a rate of 51.2% of the entire cohort displaying post-operatory HBS which was not prevented by vitamin D therapy before surgery in terms of prevalence and duration of associated intravenous therapy. However, HBS correlated, as prior mentioned, with a younger age at the moment of PTx and lower serum calcium levels before operation [107].

A large cohort (N = 796 participants with RHPT) identified a subgroup with post-surgery HBS (20.6%); pre-operatory findings were similar in terms of patients’ race and number of comorbidities (considering 31 types of associated conditions that were evaluated in this cohort) between persons who developed HBS vs. HBS-free, but the adults from the first group were younger (45.7 vs. 50.7 years, *p* < 0.001), and were more frequently obese (25% vs. 15.8%, *p* < 0.001); intra-operatory findings showed similar rates of auto transplantation (of 23%); post-operatory parameters showed that HBS correlated with a longer median hospitalization stay (of 6 vs. 3 days, *p* < 0.001) and similar readmission rates (of 23–25%) [84].

A prospective-retrospective analysis that enrolled 131 participants with RHPT who underwent PTx with self-transplantation identified a HBS rate of 76.3% and pre-operatory independent predictors of HBS were increased iPTH, bone AP, tumor total weight, and decreased serum calculated calcium [108]. A similar rate of 71.4% was revealed by the identification of HBS in 252 patients with RHPT-associated hemodialysis who underwent PTx with auto transplantation. Pre-surgery independent predictors were AP and serum (corrected) calcium of HBS [109]. A cohort of 62 consecutive persons with RHPT admitted for PTx identified a rate of 27.4% of them with HBS which correlated with younger age, increased body weight, elevated AP, and low serum calcium (as independent parameters), but did not find any association between iPTH values, neither the use of drugs such as cinacalcet or paricalcitol before PTx and HBS [110]. However, cinalcet might be found in the records of patients with chronic kidney disease before they were referred for PTx (and developed HBS), this aspect mostly indicating either an attempt to medically control the associated PT condition without PTx or a severe form of RHPT that became no responsive to medical therapy [111].

While most studies agreed that, among the bone turnover makers, AP seems the most useful in order to independently predict post-operatory HBS, a specific subgroup of individuals, especially those with normal pre-surgery AP, might benefit from the interpretation of another bone formation marker, as a close indicator of osteoblastic activity, namely osteocalcin which may serve as a surrogate for pointing the syndrome following PT removal. A study on 260 participants with RHPT referred for PTx confirmed that elevated AP, but also osteocalcin, as well as subtotal PTx and a younger age were correlated with a longer hospital stay amid HBS (according to different types of regression analyses with statistically significant results). On point, serum osteocalcin raised from a median of 264 ng/mL at baseline to 468 ng/mL following PTx (*p* < 0.001) [112]. One study showed that HBS-related bone pain positively correlated with pre-operatory PTH and post-surgery osteocalcin and negatively associated with post-PTX values of bone AP and Klotho [113].

### 3.4. Post-Operatory Findings in RHPT: Focus on HBS

As expected, HBS impairs the duration post-PTx hospitalization, readmission rate which is correlated with symptomatic electrolytes anomalies, particularly hypocalcemia, and with the need of using intravenous replacements. Early post operatory clinical and lab features of HBS are displayed from the first 18–24 h (up to one week). Intravenous calcium phase taking a few days is continued with oral calcium phase, which might take a few months. The central piece of the related biochemistry panel is calcium, but serum magnesium and phosphorus might play an important role, especially considering the longstanding kidney disease and the phosphate-associated challenging issues [114,115].

Timing of HBS onset was of 0.3 ± 0.3 months following the surgical procedure, and its duration was of 11.1 ± 14.7 months according to a study conducted between 2009 and 2019. The cohort enrolled 100 patients with PT surgery for RHPT who developed HBS and compared them with 20 controls (patients with the same diagnostic who did not experience HBS after PTx). Overall, subtotal surgical procedure was applied in 76% of cases and the others received total PTx (in addition with an auto transplantation procedure), while all individuals showed PT hyperplasia at histological examination. Post-surgery findings in persons with HBS vs. non-HBS revealed lower nadir corrected calcium, decreased (nadir and peak) iPTH, a reduced rate of persistent HPT as well as a lower rate of second (additionally needed) PTx (*p* < 0.001 for all mentioned 5 parameters). Moreover, the study identified a pre-operative parameter that functioned as predictor of HBS, namely serum ferritin that was negatively correlated with HBS (according to multivariate regression, *p* = 0.038) [4]. As mentioned by Williams-Karneskysus et al. [84], the presence of HBS doubled the hospitalization duration [84].

The largest study we identified according to our methodology was represented by a national database cohort of 7171 patients and it was published by Kravietz et al. [116] in 2018. The analysis showed that patients with PHPT (representing 58.89% of the cohort) had a 30-day readmission rate of 5.6% due to sepsis (13%), followed by hypocalcemia (12%), cardiac insufficiency (10%), and, respectively, renal anomalies (9%), while those with RHPT (21.99% of the entire group) had a higher 30-day readmission rate of 19.4% caused by hypocalcemia (22% which represented the most often complication), as well as by HBS (14%) [116].

As mentioned, early serum calcium assays after surgery are suggestive for HBS. One study highlighted a statistically significant value of lower calcium after first post-operatory 18 h in participants with HBS (27.8% of the patients from a cohort of 79 participants diagnosed with RHPT), while the only pre-operatory risk factor was younger adult age at the moment of PTx [117].

We included tertiary HPT in this analysis, particularly the unusual scenario of developing HBS after PTx in patients with pseudohypoparathyroidism. These patients displayed renal resistance to PTH and consecutive hypocalcemia, which further enhances a theoretical risk of tertiary HPT (that is actually exceptionally described) in association with PT excess-induced bone disease [118,119]. A case of pseudopypoparathyroidism type 1a was reported by Itoh et al. [119]; this was a 32-year-old male harboring a *GNAS* mutation (exon 7: c. 565_568delGACT) who developed chronic hypocalcemia-induced tertiary HPT (in association with complicated osteoporosis and brown tumors) and he experienced post-PTx HBS (with low serum calcium, phosphorus, and magnesium) requiring calcium and aphacalcidol intensive supplementation [119]. Additionally, pseudopypoparathyroidism type 1b might induce tertiary HPTH and consecutive HBS following PTx [120]. Another report introduced a 34-year-old female with X-linked hypophosphataemia (caring a c.2166delinsGG mutation) complicated with tertiary HPT and post-PTx HBS, but a consecutive normalization of renal phosphorus threshold [121]. X-linked dominant hypophosphatemic rickets is rarely complicated with tertiary HPT and the risk of further developing post-PTX HBS is rarely described [122].

Overall, our study-based analysis of reported data identified 27 studies in patients with RHPT who were referred for PTX and were then analyzed with regard to post-operatory HBS. The timeline started from 2000 to 2023 when it was published as the most recent publication. An amount of 23 studies were retrospective and 4 were of prospective, prospective–retrospective type, respectively, longitudinal and a national database cohort. Considering the number of enrolled participants per study, 12 studies included less than 100 participants per study, as follows: 19 (n = 2), 35, 37 (n = 2), 41, 45, 53, 62, 77, 79, 84; 9 studies enrolled between 100 and 200 individuals per study (of 108, 115, 120, 130, 131, 141, 148, 167, and 196), 3 studies had between 200 and 300 patients per study (specifically, 252, 260, and 297) while 3 larger studies had more than 700 patients per study (N = 796, 1846, and 7171), regardless the study design, a total of 12,468 patients with RHPT [1,4,17,30,40,84,85,104,105,106,107,108,109,110,112,116,117,123,124,125,126,127,128,129,130,131,132] (Table 5).

### 3.5. Outcome and Management in RHPT-Related HBS

An essential part of the individual decision in participants with chronic kidney disease who were referred to PTx takes into consideration local (hospital-based) protocols rather than standard specific guidelines which are currently deficient (of note, a PubMed search on “HBS” and “guideline” showed no results). Especially in individuals with chronic renal failure who are expected to display HBS with a much higher prevalence than patients with PHPT, active implementation of intra- and post-operatory protocols contribute to a better outcome. A few studies followed the results of using such protocols [123,124,125,127]. For example, one single-center study highlighted some aspects related to standard ERAS (enhanced recovery after surgery) program in patients with RHPT who were referred for total PTx without self-transplantation in order to avoid HBS. ERAS included: high-dose intravenous calcium replacement that was started immediately after surgery followed by oral supplements and calcitrol and close surveillance, as well as increased high calcium intake and dialysate. A total of 52 patients represented the group before ERAS was implemented, and 56 individuals were included in ERAS group (enrolled between 2020 and 2021), all of them being adults with RHPT under hemodialysis for at least one year. The second group developed statistically significant less frequent HBS (67.3% vs. 46.4%, *p* = 0.034). HBS correlated with a higher level of pre-operatory AP and iPTH, while post-operatory HBS associated with a longer hospital stay and a lower rate of ERAS application when compare to HBS-free participants, strongly suggesting that this type of active management is beneficial for HBS-related outcome [123].

Additionally, raising the issue of pre- and intra-operatory active interventional protocols of monitoring the patients and electrolytes replacement, we mention the longitudinal study of Ferreira et al. [124]; 77 participants diagnosed with PHPT and RHPT were enrolled before and after using the active protocol; this type of intervention increased the rate of oral calcium supplementation during post-operatory hospitalization (*p* = 0.013). HBS prevalence remained the same with patients who were protocol-free (of 9.8% in PHPT and of 58.3% in RHPT), but HBS diagnostic was not based on (clinically) symptomatic hypocalcemia as seen before protocol’s application; also, it reduced the duration of hypocalcemia (*p* = 0.047) and of hospital stay (*p* = 0.042) [124].

Another type of active intervention is represented by AP-based protocol in patients with RHPT who underwent PTx. They received calcium supplementation since the first day of surgery if pre-operatory AP was high (which correlated with a higher iPTH and a lower serum calcium before PTx, also, serving as predictors of HBS) [125].

Alternatively, an approach based on a 10-day calcitriol loading protocol before PTx in individuals with RHPT might decrease the rate of HBS, yet, it is not unanimously agreed upon. One study on 45 participants who received this mentioned type of intervention developed HBS (28.3% of them) following total PTx with auto-transplantation; HBS positively correlated with pre-operatory PTH levels, and with duration of dialysis before surgery [127]. In addition to calcium and calcitriol supplementation, the use of dialysis represents another key factor to the outcome of HBS in patients with chronic kidney disease, as similarly seen in influencing other endocrine complications of the renal condition [133,134,135]. One study showed that the rate of HBS is lower in patients with peritoneal dialysis vs. hemodialysis (75.86% vs. 92.9%, *p* = 0.004) in association with a lower total dose of intravenous calcium during early post-operatory hospitalization (*p* = 0.042), and a reduced intravenous calcium duration (*p* = 0.037) [126]. Another study showed that HBS is more frequent in patients with dialysis as opposite to transplant recipients [130].

A part from specific peri-operatory protocols of electrolytes surveillance and intervention, we should mention that certain approaches are a matter of individual decision, especially unexpected dramatic situations as, for instance, uncontrolled HBS-associated hypocalcaemia; for example, the off label use of teriparatide [136,137]. A case of tertiary HPT-associated maxillary brown tumor in USA was reported in 2020. The 48-year-old female developed HBS with severe post-PTx hypocalcemia that turned out refractory to high calcium regimes (2.5 g every 3 h) and calcitriol (2 µg twice per day) regimes, and increased hemodialysis (a total of 12 g of intravenous calcium gluconate was necessary to maintain a total serum calcium between 7 and 8 mg/dL) and teriparatide was offered (20 µg twice per day) starting with post-operatory day 25 (that was continued for one month when she was discharged with a serum calcium of 11 mg/dL) [136]. A similar case (also published in 2020) is represented by a 35-year-old woman who developed HBS following PTx for RHPT; her severe hypocalcemia persisted for 8 months remaining refractory to a standard approach, thus teriparatide was introduced (20 µg/day for the first 7 days followed by 20 µg/day, 3 times/week after dialysis) and continued for one month [137]. Teriparatide might represent an alternative to dramatic cases of HBS-associated hypocalcemia, but we still need consistent statistical evidence; yet, the drug is applied in hypoparathyroidism-associated hypocalcemia [138,139,140]. Of note, severe low total calcium levels (of 2–3 mg/dL) were associated with an increased risk of acute cardiac complications from tachyarrhythmia to asystole in association with consecutive hemodynamic instability and a potential fatal outcome [31].

At the other end of the spectrum, an aggressive correction of electrolytes anomalies underlying HBS might complicate calciphylaxis in patients with end stage renal disease who were referred for PTx [25,141]. Overtreatment with calcium and vitamin D might complicate with nephrocalcinosis and associated acute kidney failure even in patients with previously intact renal function [142].

Another uncommon complication of PTx-related HBS is represented by a pseudogout flare due to calcium-phosphate crystals [67]. Additionally, a switch from a high bone turn over to a low bone turnover following PTx within one year might be associated with an increased risk of coronary calcium score and vascular calcifications progression [128]. Yet, the presence of HBS was suggested to correlate with a bone status improvement during following months after PTX since HBS is diagnosed in patients who achieved a better control of underlying PT disease [143].

Despite a generous level of statistical evidence in RHPT according to the mentioned studies, since the topic of HBS is sometimes a matter of individual decision, we analyzed the published case reports, and identified another 25 papers with post-operatory HBS (7 cases series that enrolled 2 to 10 patients per series, and 18 single case reports, a total of 48 patients with RHPT-related HBS, aged between 23 and 74 years (these articles were published between 1989 and 2022) [8,25,26,31,111,119,120,121,122,136,137,141,143,144,145,146,147,148,149,150,151,152,153,154,155] (Table 6).

## 4. Discussion

Of historical note, Fuller Albright was the first to report HBS following PTx in addition to other major contributions he made to the development and understanding of parathyroid and bone field [156,157,158,159]. The oldest report of HBS after PT surgery (according to our method of PubMed research) is from 1976 (a PHPT case complicated with hypocalcemia- and hypomagnesaemia-associated congestive heart failure) [83]. One of the most impressive pediatric case of HBS was reported in 1998, a 6-year-old girl who developed neurological anomalies since having hypercalcemia (detected within her first days of life); the total serum calcium was one the highest at such young age (of 25.5 mg/dL) in association with increased PTH (of 1550 pg/mL) [90]. Nowadays, HBS still represents a challenge after PT removal, but otherwise, PT surgery offers an impressive curative rate of underlying PT disorders [160,161]. Our case sample-based study, to our knowledge, is the largest of its kind, embracing the scientific work published over time (N = 14,349 subjects with PHPT and RHPT), according to our methodology of research.

### 4.1. Panel of Investigations in PHPT and RHPT as Clues for HBS

RHPT associates a higher risk of developing HBS as compared to PHPT. HBS prevalence varies from 15% to 25% up to 75–92% in RHPT, while in participants with PHPT mostly one out of five adults, respectively, one out of three children and teenagers might be affected by the syndrome (if any, depending on study).

In PHPT, we observed four clusters of potential HBS indicators. The first one (and probably the most important) is represented by pre-operatory panel of biochemistry and endocrine parameters, especially, increased PTH [35,39,40,43,46] and increased AP [35,43,46,78]. Additionally, in this category, we mention makers of associated renal damage or elevated blood urea nitrogen [34,43,46], and, probably, a very high serum calcium [46]. The second category comes from the clinical presentation, meaning an older age for adults (yet, not all authors agree) [37,46]; particular skeleton involvement (level of case reports) especially brown tumors and osteitis fibrosa cystica [7,20,56,57,58,65,69,70,75,77,88,95,96]; less than convincing evidence includes the presence of osteoporosis [43] or of a PT crisis [48,71,78]. The third category comes from the PT tumor itself; larger adenomas might have a higher risk [37,46] or tumors with an increased weight [39,82], giant PT adenomas [50,51,78,88], ectopic adenomas [68,69,71,79,80,86], PT carcinomas [52,54,55,65,76,81], atypical PT tumors [10,48,96]. The fourth category is based on the intra-operatory and early post-surgery management, meaning an associated thyroid surgery [37,39,42,43] and, maybe, a prolonged PTx time (but this is still an open issue) [39] increases the risk, as opposed to prompt recognition of HBS based on calcium (and PTH) assays and rapid intervention (specific interventional protocols are rather used in RHPT than in PHPT) [41]. Two important aspects are not clarified yet: the use of pre-operatory bisphosphonates [14,42,45] and the usefulness of 25-hydroxyitamin D assessment as a predictor of HBS which is less proved so far [37,40].

**Figure 2:**
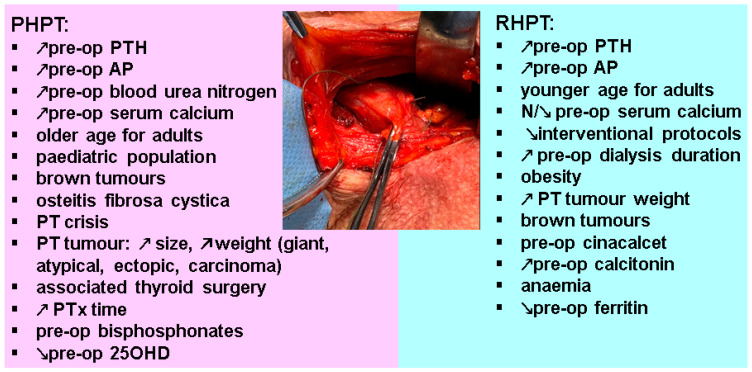
Qualitative analysis of clinical, lab, and imaging parameters that may serve as pointers of HBS following PTx in patients with PHPT and RHPT [1,4,6,17,30,34,35,36,37,38,39,40,41,42,43,44,45,46,84,85,104,105,106,107,108,109,110,112,116,117,123,124,125,126,127,128,129,130,131,132]. Abbreviations: PHPT = primary hyperparathyroidism; RHPT = renal hyperparathyroidism; pre-op = pre-operatory; PTH = parathyroid hormone; AP = alkaline phosphatase; PT = parathyroid; PTx = parathyroidectomy; 25OHD = 25-hydroxyvitamin D.

With regard to RHPT, we mention three types of evidence. The first includes risk factors for HBS with a solid level of statistical evidence: younger age at PTx [84,85,105,107,110,112,117,130], pre-operatory elevated level of AP [85,104,105,108,110,112,123,130], and PTH [85,104,105,108,123,127,130], respectively, normal/low serum calcium [85,105,107,108,110]. The second group includes active interventional protocols that either reduce the rate or improve the severity of HBS, in addition to an adequate use of dialysis during early post-operatory stages [123,124,127]. The third category involves data with inconsistent evidence that might be the objective of future studies to a better understanding; for instance, a longer pre-surgery dialysis duration [104,127], obesity [84,110], a higher total tumor weight [108], an elevated pre-surgery calcitonin [104], prior use of cinalcet [111], the co-presence of brown tumors and osteitis fibrosa cystica as seen in PHPT [137,148], *GNAS* mutation [119] (Figure 2).

On a larger picture, we mention some aspects that are still a matter of debate. Park-Sigal et al. [148] reported a case of PHPT with multiple complications that associated primary hyperaldosteronism; we do not have enough evidence to correlate it with HBS [148]. Some studies included patients with PHPT associated with renal damage, but not representing secondary (renal) or tertiary HPT, and this aspect might embrace the scenario of RHPT following PTx [43]. Paepegaey et al. [66] suggested that intense bone uptake at F-fluorocholine positron emission tomography/computed tomography represents a clue for post-PTx HBS in PHPT, but we currently do not have enough data to explore this tool [66]. PT crisis, also named “hyperparathyroid crisis” was reported in some patients who further developed HBS; this severe clinical entity typically includes arrhythmia-related palpitations, dyspnea, fatigue, nausea, and rapid weight loss; yet, the current use of the term is limited on daily basis. Thus, the actual clinical utility as a pointer of HBS is debatable [49,71].

Anemia (mainly of normocytic normochromic type in PHPT) is found in long standing PHPT due to direct PTH inhibition on erythrocytes, increased peripheral calcium-mediated destruction of red cells, long-term high PTH-induced medullary fibrosis while anemia in chronic kidney disease, a part form indicating a long standing severe condition, correlates with lack of kidney-associated erythropoietin synthesis [7,26,109]. A combination of PA, iPTH, and hemoglobin might serve as a prediction model of post-operatory serum calcium levels and different aspects of calcium regimes [109]. Ferritin as a pre-PTx predictor of HBS in RHPT was identified in one study [4].

Pulmonary embolism (according to isolated reports) might be related to chronic hypercalcemia rather than an elevated PTH level underling a high calcium-induced pro-inflammatory and hyper-coagulation status, mechanisms that are yet to be determined [7,65]. Additionally, PT carcinomas are prone to multiple thromboses in advanced stages [65].

A randomized study conducted by Salman et al. [37] highlighted that participants with HBS had lower levels of 25-hydroxyvitamin D vs. those HBS-free, but the assay itself did not serve as an independent HBS predictor, neither did active vitamin D supplementation prevent HBS [37]. We need more randomized, interventional studies in order to address the issue of vitamin D supplementation before PTx with respect to HBS risk, as opposite to well established data we already have concerning non-HBS benefits of careful cholecalciferol administration before surgery in patients with PHPT [37,64,162].

Additionally, the presence of brown tumor represents an aggravating factor for HBS according to the reports we have so far for this exceptional skeleton finding [7,20,56,57,58,65,69,70,75,77,88,95,96]. Direct osseous access for a histological confirmation is usually done in patients with a high index of malignancy suspicion or in cases complicated with pathological fractures requiring an open orthopedic intervention. However, in the absence of bone biopsy, serial imaging follow-up is mostly useful since a successful PTx associates a remission of the skeleton tumors [7,77]. Whether HBS correlates with their remission rate is yet to be determined.

### 4.2. Surgical Procedures

PTx in terms of surgical procedures might influence the outcome of HBS. A study on 297 participants with RHPT showed a rate of 31.3% in patients who underwent subtotal PTx, and of 6.9% following incomplete PTx (*p* = 0.006) [129]. Incomplete PT adenomas resection or post-PTx persistent HPT might serve as clues of not developing HBS; however, HBS is described in cases where PTH levels decreased, but not becoming low as seen in post-PTx hypoparathyroidism [51]. Another interesting scenario was reported in a case with RHPT with a history of two subtotal PTxs in addition to long time hemodialysis who actually developed HBS after a consecutive renal transplant [146].

We currently do not have enough data to sustain if ectopic location such as a mediastinal PT adenoma associates a higher risk of post-operatory HBS than orthotopic PT tumors despite the more complicated intervention according to cardiothoracic surgical protocols [52,71]. Similarly, the evidence concerning other types of PTx approaches is inconsistent, as, for instance, the use of trans-oral endoscopic parathyroidectomy vestibular approach (m-TOEPVA) [144]. Ectopic PT adenoma in patients with RHPT may associate persistent or recurrent HPT after PTx; in this situation, the risk of developing HBS after a second intervention is probably higher [145]. Thoracoscopic resection for ectopic PT adenoma represents a cutting age approach in pediatric population, and post-operatory HBS was identified, as seen in adults [76,97]. Robot-assisted thoracic surgery for mediastinal PT adenoma was reported to be followed by HBS in a case with PHPT complicated with osteitis fibrosa cystica [72].

Additionally, the association of thyroidectomy of various types that is synchronously performed with PT surgery was found to increase the risk of HBS according to the study of Guillén Martínez et al. (N = 82 patients with PHPT) and of Jakubauskas et al. (N = 94 participants with PHPT), respectively [41,42]. This represents an important aspect to be taken into consideration noting the high prevalence of thyroid nodules in general population [163]. A mostly unusual case of multiple endocrine neoplasia type 1 included a concomitant active PHPT with Basedow–Graves’ disease and acromegaly; the 54-year-old woman underwent concomitant PTx and thyroidectomy and consecutively developed HBS [77].

### 4.3. Individual Decision or Protocol-Based Management in HBS

Prompt intervention is advised in HBS, and some hospital-based protocols specify an active intervention immediate after PTx especially in cases with RHPT. An elemental calcium dose of 4–12 g per day, first intravenous and then oral, in addition to 2–4 µg of calcitriol and, potentially, magnesium supplements are necessary. Persistent severe hypophosphatemia, an exceptional component of HBS after surgery for RHPT, is more difficult to be treated than long term hypocalcemia [12]. Frajewicki et al. [153] reported more than three decades ago intraperitoneally administration of phosphate in a case of post-PTx HBS for RHPT [153].

Other pro-active measurements include adequate vitamin D therapy before PTx and even anti-resorptive medication to reduce hypercalcemia, and, potentially, to control HBS, but not all authors agree with a clear reduction in HBS prevalence and/or severity [7]. Bisphosphonates were reported to improve HBS by some authors [13,20,45,48]. Davenport et al. [20] described a study on pamidronate to reduce the rate of HBS in RHPT [20]. Lee et al. [48] concluded that this class of medication may be beneficial for HBS in PHPT [48]. On the contrary, Zelano et al. [68] found that a more severe form of HBS, as was confirmed in their reported PT carcinoma case, was partially due to the short pre-operatory course of bisphosphonates [68]. As suggested by Corsello et al. [22], the medication is responsible for reducing excess PTH-associated bone resorption, but the limited amount of time concerning the drug exposure seems not enough to allow a coupled decreased in bone formation [22,68]. Additionally, another retrospective cohort (N = 19 patients with PHPT) showed that none of the 11 participants who received zolendronic acid (a dose of 4 mg within 1 or 2 days before PTx) developed HBS, while 3/8 individuals who did not receive the drug experienced HBS [45].

### 4.4. Differential Diagnostic of Post-PTx Hypocalcemia

**Figure 3:**
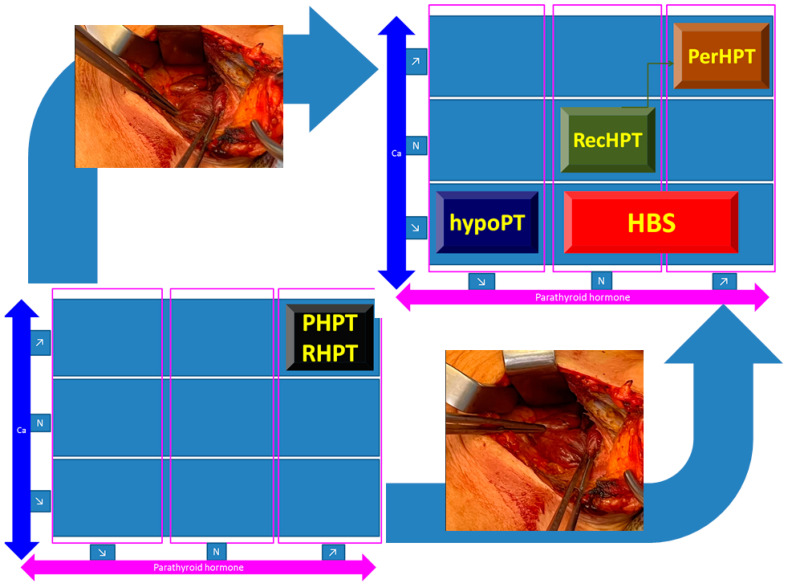
The outcome after PTx ((**right**) side) in patients diagnosed with PHPT and RHPT ((**left**) side) regarding different combinations of calcium and PTH assays: hypoparathyroidism (low calcium and PTH), HBS (low calcium with non-low PTH), persistent HPT (high calcium and PTH), recurrent HPT (normal calcium and PTH) followed by a relapse of the elevated calcium and PTH values [164,165]. Abbreviations: Ca = serum calcium levels; N = normal; ↗ = increased values; ↘ = low values; PHPT = primary hyperparathyroidism; RHPT = renal hyperparathyroidism; PerHPT = persistent hyperparathyroidism; RecHPT = recurrent hyperparathyroidism; hypoPT = hypoparathyroidism; HBS = hungry bone syndrome (Both captures are intra-operatory aspects during PTx of a right inferior PT adenoma on an adult diagnosed with PHPT).

Hypoparathyroidism (low calcium and low PTH) represents a more frequent complication/outcome after PTx and it is the main differential diagnostic of HBS [164,165] (Figure 3).

Post-PTx acute bone pain in individuals with HBS might be differentiated from other post-operatory complications such as chrondrocalcinosis due to sudden drop of calcium and magnesium [7]. Older reports suggested that post-PTx-associated transitory thyrotoxicosis due to gland manipulation might mimic HBS [166].

### 4.5. Integrating PTx-Related HBS to Non-PTx Causes of HBS

Thyroidectomy exposes the patients to HBS to a much lesser degree than seen in PTx. One mechanism of serum calcium fall, especially if the patient experienced thyrotoxicosis-associated hypercalcemia, is increased osteoblastic activity, bone formation being more exacerbated than bone resorption after normalization of thyroid status. Excessive thyroid hormones cause an elevation of osteoblast-derivate bone turnover favoring the resorption while acute drop of these hormones causes a prompt reversal of bone resorption, newly synthesized osteoid being “hungry” to deposit calcium which leads to hypocalcemia [167,168,169,170,171]. Lazareva et al. reported a teenager with Basedow’s disease who developed HBS after radioiodine ablation [171]. Post-thyroidectomy hypocalcemia, on the other hand, may be caused by transitory or permanent hypoparathyroidism, being described with a much more important epidemiological impact than other complications [172].

Cinacalcet might induce a pharmacologic PTx [173,174,175]. A report from 2018 (the most recent according to our methods of search) introduced a patient with RHPT treated with the drug who developed cinacalcet-induced HBS after 2 weeks since starting the medication and remitted 4 weeks after stopping it under therapy with calcium and vitamin D [173]. The first two published cases we were able to identify regarding cinalcet-derivate HBS date from 2006 and 2007, respectively, both in patients with RHPT [176,177]. Alternatively, pre-operatory use of cinalcet was suggested to play a certain role in improving post-PTx HBS [150]. Recently (in 2022), a denosumab-induced HBS-like was reported [178].

In 2021, the first case of HBS following a living donor hepatic transplant was reported in a 5-month-old child diagnosed with biliary atresia; the boy had craniotabes before the operation and associated HBS for 1 month after transplant (requiring calcium and vitamin D replacements) followed by rapid elevation of bone specific AP and craniotabes improvement. The most probable mechanism is increased bone metabolism due to restauration of liver function [179].

Another most challenging etiological form of HBS is reported after the removal of a mesenchymal tumor with over production of Fibroblast Growth-Factor-23 (FGF-23) with phosphaturic effect and causing tumor-induced osteomalacia [180,181]. A more known cause of non-PTx-associated HBS relates to osteoblastic metastasis from prostate (majority of cases) or gastric cancers (exceptionally) [182,183,184,185].

Additionally, at single case report level, we mention a 35-year-old woman who experienced a markedly elevated hypercalcemia (of 21 mg/dL) with suppressed PTH while being pregnant (32 weeks of gestation). After cesarean, she had a transitory HBS which was considered to be caused by a placental PTHrP-related hypercalcemia [186]. Another hypothesis of non-PTx HBS is related to bisphosphonates therapy for Paget’s disease of the bone characterized by increased bone turnover, while acute suppression of resorption via risendronate, as was used in one case, might favor to continuing the bone formation [187].

Finally, according to presented data, we are not aware of a larger analysis on published cases concerning HBS (N = 14,349). We chose not to use a systematic review in order to include a larger area of particular aspects in HBS, many of them not being covered by a high level of statistical evidence. With regard to PHPT, we identified 14 studies (N = 1545 patients, a maximum 425 participants per study), and 36 case reports (N = 37), a total of 1582 adults, aged between 20 and 72. Concerning pediatric studies in PHPT, we found three of them (N = 232 children and teenagers, a maximum of 182 participants of studied population per cohort), and 15 case reports (N = 19), a total of 251 patients, aged between 6 and 18. RHPT and HBS was studied in 27 studies (N = 12,468 individuals, the largest cohort of 7171) and in 25 case reports and series (N = 48), a total of 12,516 persons, aged between 23 and 74.

## 5. Conclusions

HBS remains a rare complication following PTx, yet extremely severe and with a certain level of predictability; thus, the importance of being adequately identified and managed. The pre-operatory spectrum of assessments is based on biochemistry and hormonal panel in addition to a specific (mostly severe) clinical presentation, while the PT tumor itself might provide useful insights concerning the size and the proliferation profile as potential risk factors. Particularly in RHPT, where the reported rates of HBS were higher than in PHPT, prompt interventional protocols of electrolytes surveillance and replacement, despite not being yet a matter of a unified, specific guideline, prevent symptomatic hypocalcemia and reduce the hospitalization stay and the re-admission rates.

## Figures and Tables

**Table 1:** Original studies in adult individuals diagnosed with PHPT submitted to PTx and complicated with post-operatory HBS; the display starts with the most recent publication timeline [6,34,35,36,37,38,39,40,41,42,43,44,45,46].

First Author Year of Publication Reference Number	Study Design Studied Population	Post-Operatory Outcome (HBS)
Tang 2022 [34]	Retrospective study (between 2000 and 2018) N = 28 adults with atypical PT adenoma + PHPT mean age: 56 y 1/3 with renal dysfunction and stones 1/5 with bone loss	HBS prevalence: 7%
Chandran 2022 [35]	Retrospective study (between 2012 and 2019) N = 164 adults with PHPT	HBS prevalence: 2.4% Pre-operatory predictors of HBS: iPTH and AP
Nouikes Zitouni 2021 [36]	Retrospective study (between 2002 and 2013) N = 62 adults with PHPT mean age: 47 y	HBS prevalence: 19.3%
Salman 2021 [37]	Interventional study N = 102 p with PHPT N1 = 52 p without VD replacements N2 = 25 p with VD replacements + 25OHD > 20 ng/mL N3 = 25 p with VD replacements + 25OHD > 20 ng/mL	HBS prevalence: 8%, 16%, 23% (p = NS) Pre-operatory 25OHD is not an independent predictor of HBS.
Guillén Martínez 2020 [38]	Case-control, observational, analytical study (between 2007 and 2016) N = 82 p with PHPT	HBS prevalence: 12.2% HBS correlated with: 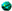 thyroid surgery 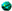 older age (>68 y) 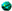 PT tumor diameter > 1.7 cm
Jakubauskas 2018 [39]	Retrospective study (between 2005 and 2016) N = 94 p with PHPT	HBS prevalence: 28.7% HBS correlated with: 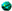 thyroid surgery 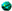 PT tumor weight 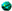 iPTH 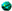 prolonged time of PTx
Kaderli 2018 [40]	Retrospective study N = 385 p with PHPT	HBS prevalence: 8.6% Pre-operatory predictor: PTH (not 25OHD)
Kaderli (bis) 2018 [41]	Retrospective study N = 425 p with PHPT	PTH in post-operatory day 1: discriminative for transitory hypoPT, not for HBS PTH in post-operatory day 5–7: diagnostic of HBS
Mayilvaganan 2017 [42]	Retrospective study (between 2013 and 2015) N = 19 p with PHPT N1 = 11 p with pre-PTx ZOL N2 = 8 p without pre-PTx ZOL	HBS prevalence: N1 = 0/11 N2 = 3/8
Kaya 2016 [43]	Retrospective study N = 62 p with PHPT	HBS prevalence: 13.4% HBS correlated with: 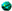 thyroid surgery 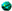 PT tumor hyperplasia 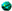 iPTH 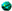 AP 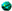 blood urea nitrogen 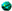 osteoporosis
Prasarttong-Osoth 2012 [6]	Retrospective study (between 1997 and 2007) N = 45 p with PHPT	HBS prevalence: 22%
Malabu 2007 [44]	Retrospective study (between 200 and 2006) N = 46 p with PHPT	HBS prevalence (+recurrent HPT): 4%
Lee 2006 [45]	Retrospective study (between 1997 and 2002) N = 23 p with PHPT	HBS: 9/23 (BP exposure: 0/9) HBS-free: 14/23 (BP exposure: 6/14)
Brasier 1988 [46]	Retrospective study N = 219 p with PHPT	HBS prevalence: 12.6% HBS correlated with: 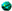 older age (by mean 10 y) 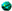 ↗serum calcium 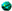 ↗AP 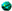 ↗PTH 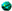 ↗blood urea nitrogen 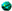 larger PT adenoma

Abbreviations: AP = alkaline phosphatase; PTH = parathormone; HBS = hungry bone syndrome; BP = bisphosphonates; PT = parathyroid; PHPT = primary hyperparathyroidism; PTx = parathyroidectomy; p = patients; hypoPT = hypoparathyroidism; VD = vitamin D; 25OHD = 25-hydroxyvitamin D; NS = non-significant; N = number of patients; ZOL = zoledronic acid; y = year.

**Table 2:** Original case reports in adults diagnosed with PHPT referred to PTx and complicated with HBS; the display starts with the most recent publication timeline [7,10,11,14,15,19,20,48,50,51,52,53,54,55,56,57,58,65,66,67,68,69,70,71,72,73,74,75,76,77,78,79,80,81,82,83].

First Author Year of Publication Reference Number	Presentation	Post-Operatory Outcome (HBS)
Shah 2023 [14]	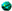 64-y male with PHPT 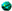 5.3 g PT adenoma	Pre-operatory: 2 rounds of hemodialysis + calcitonin + ZOL
Zelano 2022 [65]	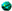 56-y male with PHPT and PT carcinoma 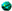 complications: portal thrombosis, pancreatitis, brown tumors, chrondrocalcinosis, depression	HBS (IV therapy for 4 days, oral therapy for 8 months)
Landeta 2022 [48]	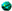 56-y male with PHPT and atypical PT adenoma 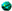 admission for parathyroid crisis	HBS for 6 months after en bloc resection
Alvarez-Payares 2022 [7]	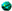 55-y female with PHPT and giant PT adenoma (of 5 cm, 16 g) 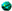 admission with OFC, brown tumors and anemia	Post-operatory HBS since first 72 h (complicated with pulmonary embolism)
Parikh 2021 [58]	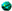 30-y female with PHPT 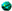 multiple brown tumors at spine and pelvis	Total PTX followed by HBS
Raj 2020 [51]	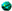 66-y female with giant PHPT 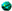 PT adenoma of 28.7 g	HBS (IV therapy for 4 weeks)
Buisset 2019 [54]	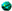 20-y female with PHPT and PT carcinoma	HBS (+right thyroid lobectomy)
Florakis 2019 [10]	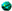 44-y male with PHPT 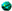 atypical PT adenoma	HBS: 2-day hospitalization 6-month post-PTx HBS
Paepegaey 2019 [66]	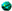 57-y male with PHPT 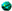 intense bone F-fluorocholine PET/CT	HBS (as indicated by PET/CT)
Tai 2018 [67]	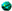 56-y female with PHPT	HBS complicated with pseudogout flare
Schnyder 2017 [20]	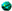 72-y female with PHPT 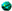 brown tumor mimicking metastasis from a previous breast cancer	HBS as clue for OFC, not bone metastasis
Rutledge 2016 [50]	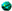 21-y female with PHPT 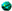 giant PT adenoma (of 59 g, of 8 cm)	HBS since post-operatory day 3
Zhou 2016 [68]	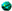 67-y male with PHPT and ectopic PT adenoma	HBS
Irie 2015 [56]	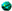 31-y female with PHPT and patellar brown tumor	HBS (IV therapy for 30 days) Patellar pain resolution 1 month since PTx.
Sridhar 2014 [69]	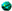 N = 1 p with PHPT and mediastinal PT adenoma and osteitis fibrosis cystica 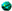 robot-assisted thoracic surgical resection	HBS (the patient had severe obesity)
Varma 2014 [70]	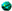 36-y female with PHPT and iliac brown tumor	HBS
Gratian 2014 [71]	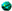 60-y female with PHPT 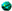 onset as hyperparathyroid crisis 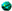 PT hyperplasia and ectopic PT gland	HBS
Rastogi 2013 [72]	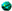 30-y female with PHPT + PT adenoma and pseudoarthrosis 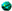 undetectable pre-operatory 25OHD	HBS
Ohe 2013 [52]	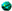 N = 2 p with PT carcinoma	HBS
Wang G 2013 [73]	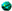 20-y male with PHPT and ectopic PT (thymus) 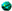 severe 25OHD deficiency	HBS
Tachibana 2012 [74]	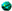 54-y female with MEN1 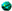 concomitant PHPT + BD + acromegaly	PTx and thyroidectomy → HBS
Kim 2012 [53]	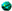 29-y female with minimally invasive PT carcinoma	HBS
Silaghi 2011 [75]	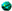 48-y female with PHPT and brown tumors	HBS
Corsello 2010 [19]	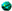 64-y female with PHPT	HBS (ZOL before PTx)
Yong 2010 [55]	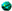 23-y male with PHPT and PT carcinoma	HBS
Sandoval 2010 [11]	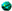 63-y female with PHPT	HBS (IV therapy since day 7 to day 15)
Ajmi 2010 [57]	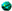 48-y female with PHPT and brown tumors	HBS
Rathi 2008 [76]	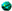 45-y female with PHPT and PT carcinoma	HBS
Meydan 2006 [77]	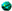 52-y female with PHPT and brown tumors	HBS
Morrone 2005 [15]	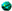 55-y male with PHPT and very high AP	HBS (AP dynamics reflects calcium changes)
Kuzucu 2002 [78]	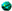 N = 1 p with PHPT and giant PT adenoma 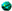 hyperparathyroid crisis	HBS
Chandran 2003 [79]	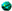 33-y female with PHPT and thymic PT carcinoma	HBS
Chen 1996 [80]	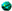 20-y female with PHPT and ectopic PT adenoma	HBS
Liou 1996 [81]	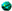 64-y male with PHPT and PT carcinoma 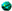 chronic kidney failure to renal stones	Post-PTx → early asymptomatic hypocalcemia → symptomatic HBS since month 8 (+↗PTH)
Natsui 1996 [82]	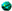 29-y male with PHPT 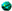 PT adenoma of 8.5 g	HBS
Falko 1976 [83]	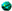 N = 1 with PHPT	HBS complicated with congestive heart failure

Abbreviations: AP = alkaline phosphatase; BS = Basedow’s disease; HBS = hungry bone syndrome; hypoPT = hypoparathyroidism; IV = intravenous; MEN = multiple endocrine neoplasia; PT = parathyroid; PHPT = primary hyperparathyroidism; PTx = parathyroidectomy; p = patients; PET/CT = positron emission tomography/computed tomography; VD = vitamin D; 25OHD = 25-hydroxyvitamin D; OFC = osteitis fibrosa cystica; NS = non-significant; N = number of patients; ZOL = zoledronic acid; y = year.

**Table 3:** Original studies in children and adolescents diagnosed with PHPT and analyzing HBS after PTx; the display starts with the most recent publication timeline [91,92,93].

First Author Year of Publication Reference Number	Study Design Studied Population	Post-Operatory Outcome (HBS)
Sharanappa 2021 [91]	Retrospective study (between 1989 and 2019) N = 35 p with PHPT (<18 y) mean age: 15.2 y	HBS prevalence: 35%
Hanba 2016 [92]	Retrospective pediatric study (between 2009 and 2012) N = 182 p with N’ = 262 PTx	Post-operatory prolonged hospital stat correlated with: 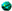 male sex 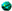 younger age than ≤15 y 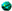 renal damage
George 2010 [93]	Retrospective pediatric study (between 1993 and 2006) N = 15 p with PHPT	HBS prevalence: 33.3%

Abbreviations: HBS = hungry bone syndrome; PHPT = primary hyperparathyroidism; PTx = parathyroidectomy; N = number of patients; y = year; p = patients.

**Table 4:** Original case reports/series of pediatric PHPT analyzing post-PTX HBS; the display starts with the most recent publication [86,87,88,89,90,91,92,93,94,95,96,97,98,99,100,101,102,103].

First Author Year of Publication Reference Number	Presentation	Post-Operatory Outcome (HBS)
Boro 2022 [94]	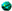 N = 10 teenagers with PHPT 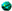 typical/atypical adenoma = 9/1	HBS prevalence: 40% (N = 4)
Vitale 2022 [97]	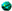 12-y male with ectopic PT adenoma 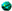 complications: slipped capital femoral epiphysis 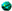 associating obesity and autism	HBS (pediatric thoracoscopic resection)
Tuli 2021 [87]	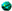 N = 2 p with pediatric PHPT 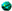 1 case treated with cinalcet	HBS (non-cinalcet case)
Legault 2020 [95]	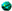 14-y male with PHPT and giant PT adenoma 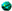 pelvic brown tumor	HBS (IV therapy of 6 days)
Lenherr-Taube 2020 [88]	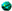 13-y male with PHPT and PT carcinoma and brown tumors	HBS (IV therapy for 3 weeks)
Hendarto 2017 [89]	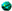 18-y female with PHPT 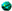 multiple fractures and scoliosis	HBS
Juárez-León 2015 [96]	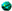 18-y female with PHPT and brown tumors 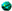 atypical PT adenoma (of 3.8 g)	prolonged HBS (for 42 months: calcium supplements)
Saif 2015 [98]	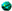 15-y female with PHPT 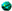 onset as acute pancreatitis	HBS (IV therapy for 5 days)
Ebina 2015 [99]	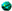 16-y male with PHPT 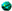 onset with osteolytic fractures of femoral neck and radial shaft	HBS (IV therapy from day 4 to 28 after PTx)
Çelik 2014 [100]	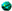 N = 5 p with PHPT 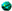 mean age: 11 y	1/5 developed HBS
Yeşilkaya 2009 [86]	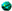 12-y female with PHPT 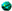 ectopic PT adenoma	HBS
Simsek 2009 [101]	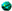 10-y female with PHPT	HBS (IV therapy for 4 weeks)
Damiani 1998 [90]	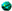 6-y female with PHPT (starting with day 8 of life)	HBS
Boechat 1996 [102]	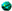 N = 2 p with pediatric PHPT	HBS
Kale 1992 [103]	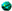 15-y child with PHPT	HBS

Abbreviations: HBS = hungry bone syndrome; g = grams; IV = intravenous; PHPT = primary hyperparathyroidism; PTx = parathyroidectomy; PT = parathyroid; N = number of patients; y = year; p = patients.

**Table 5:** Original studies in patients confirmed with RHPT who developed HBS following PTx; the display starts with the most recent publication [1,4,17,30,40,84,85,104,105,106,107,108,109,110,112,116,117,123,124,125,126,127,128,129,130,131,132].

First Author Year of Publication Reference Number	Study Design Studied Population	Post-Operatory Outcome (HBS)
Tai 2023 [4]	Single-center, retrospective study (between 2009–2019) N = 120 p with RHPT + PTx N1 = 100 p + ve HBS N2 = 20 p HBS free	Pre-operative predictor for HBS: serum ferritin (*p* = 0.038) Post-operative parameters: N1 < N2 (*p* < 0.001) nadir corrected Ca nadir and peak iPTH rate of persistent HPT rate of second PTx
Tanweer 2023 [30]	Single-surgeon experience (between 2016 and 2020) N = 53 p with PTx mean age: 75 y	3.7% with post-operative HBS (among RHPT)
Wang L 2022 [123]	Single-center, retrospective study (between 2020 and 2021) N = 108 p with RHPT N1 = 52 p (no ERAS program) N2 = 56 p (ERAS program)	Pre-operatory predictor for HBS: ↗AP ↗PTH HBS prevalence:N1 = 46.4% vs. N2 = 67.3% (*p* = 0.034) Post-operatory HBS correlated with: longer hospital stay lower rate of ERAS application
Peng 2022 [104]	Single-center, retrospective study (between 2015 and 2021) N = 141 p with RHPT + successful PTx N1 = 46 p with HBS (32%) N2 = 95 p HBS free	Pre-operatory predictor for HBS: N1 > N2 (*p* < 0.05) dialysis age PTH Calcitonin AP
Williams-Karnesky 2022 [84]	Retrospective study N = 796 p with RHPT + PTx N1 = 164 p with HBS (20.6%) N2 = 632 p HBS free	Pre-operatory predictor for HBS: younger age more frequent obesity Post-operatory HBS correlated with: longer hospital stay
Ferreira 2021 [124]	Retrospective study N = 77 p with PHPT and RHPT	Active monitoring and electrolytes replacement consequences: similar HBS rate but reduced severity (9.8% in PHPT and 58.3% in RHPT) reduced hypocalcemia duration (*p* = 0.047) reduced hospital stay (*p* = 0.042)
Kritmetapak 2021 [85]	Retrospective study (between 2014 and 2020) N = 130 p with RHPT + PTx N1 = 85.4% with HBS	Pre-operatory predictor for HBS: younger age (≤45 y) AP (>420 U/L) iPTH (>1000 pg/mL) normal calcium (<10.2 mg/dL) Post-operatory HBS correlated with: longer hospital stay
Stefanova 2020 [1]	Retrospective study (between 2011 and 2016) N = 1846 p with RHPT + PTx	HBS and hypocalcemia caused 47% of post-PTX readmissions.
Wang M 2020 [108]	Prospective—retrospective study (between 2016 and 2018) N = 131 p with RHPT + PTx	HBS prevalence: 76.3% Pre-operatory independent predictors: ↗iPTH ↗AP ↗tumor total weight ↘calculated serum calcium
Wong 2020 [125]	Retrospective study (between 2008 and 2013) N = 167 p with RHPT + PTx	Hypocalcemia (including HBS) rate: 10.9% Pre-operatory AP-based calcium supplementation for HBS
Ko 2020 [112]	Retrospective study (between 2010 and 2017) N = 260 p with RHPT + PTx	Pre-operatory predictors for HBS: younger age ↗AP ↗osteocalcin subtotal PTx
Ge 2020 [105]	Retrospective study (between 2015 and 2017) N = 115 p with RHPT + PTx	HBS prevalence: 87.8% Pre-operatory predictors for HBS occurrence: ↗AP ↘serum calcium Pre-operatory predictors for HBS severity: ↗AP ↗iPTH younger age
Yang 2019 [126]	Retrospective study N1 = 169 p with RHPT + PTx + HD N2 = 29 p with RHPT + PTx + PD	HBS prevalence: N1 = 92.9% N2 = 75.86% (*p* = 0.004)
Ferreira 2019 [127]	Retrospective study N = 45 p with RHPT + PTx + 10-day calcitriol protocol before PTX	HBS prevalence: 28.3% Pre-operatory predictors for HBS: iPTH duration of dialysis
Yang 2018 [109]	Retrospective study N = 252 p with RHPT + PTx + hemodialysis	HBS prevalence: 71.4% Pre-operatory predictors for HBS: PA serum (corrected) calcium
Fülöp 2018 [106]	Retrospective study (between 2005 and 2016) N = 37 p with RHPT + PTx	Younger age correlated with: post-PTx hypocalcemia post-PTx duration of hospitalization
Kravietz 2018 [116]	National database study (between 2013 and 2014) N = 7171 p with PTx N1 = 58.89% with PHPT N2 = 21.99% with RHPT	30-day readmission rate due to: N1: sepsis (13%), hypocalcemia (12%) N2: hypocalcemia (22%), HBS (14%)
Schneider 2018 [40]	Prospective observational pilot study (between 2010 and 2012) N = 35 p with RHPT	HBS-associated bone pain correlated with: iPTH and osteocalcin (pre-operatory) AP and Klotho (post-operatory)
Hernandes 2017 [128]	Follow-up study N = 19 p with RHPT	After 6-month HBS → at 12 months: low bone turnover associated with vascular calcifications progression
Ho 2017 [110]	Retrospective study N = 62 p with RHPT	HBS prevalence: 27.4% Pre-operatory predictors for HBS: ↗PA young age ↗ body weight ↘serum calcium
Konturek 2016 [129]	Retrospective study (between 1995 and 2014) N = 297 p with RHPT	HBS prevalence: 31.3% (subtotal PTx) vs. 6.9% (incomplete PTx)
Florescu 2014 [130]	Retrospective study N = 41 p with RHPT N1 = 73% under dialysis N2 = 27% renal transplant	Pre-operatory predictors for HBS: PA iPTH young age Post-operatory correlation with HBS: level of PTH decrease
Latus 2013 [107]	Retrospective study N = 84 p with RHPT	HBS prevalence: 51.2% Pre-operatory predictors for HBS: young age ↘serum calcium
Goldfarb 2012 [117]	Retrospective study N = 79 p with RHPT	HBS prevalence: 27.8%Pre-operatory predictor for HBS: young age
Davenport 2009 [17]	Retrospective study N = 37 p with RHPT	Pre-operatory use of pamidronate 24–48 h before PTx (27/37) → HBS (2/27)
Jofré 2003 [131]	Retrospective study N = 148 p with RHPT	HBS prevalence: 20%
Zhong 2000 [132]	Retrospective study (between 1994 and 1998) N = 19 p with RHPT	HBS prevalence: 15.78%

Abbreviations: AP = alkaline phosphatase; Ca = calcium; ERAS = enhanced recovery after surgery; HBS = hungry bone syndrome; HPT = hyperparathyroidism; HD = hemodialysis; iPTH = intact parathormone; PD = peritoneal dialysis; p = patient; PTx = parathyroidectomy; RHPT = renal hyperparathyroidism.

**Table 6:** Original case reports and series in patients diagnosed with RHPT referred to PTx and complicated with HBS; the display starts with the most recent publication timeline [8,25,26,31,111,119,120,121,122,136,137,141,143,144,145,146,147,148,149,150,151,152,153,154,155].

First Author Year of Publication Reference Number	Presentation	Post-Operatory Outcome (HBS)
Itoh 2022 [119]	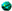 32-y male with tertiary HPT 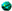 *GNAS* mutation 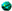 osteoporosis and brown tumors	Post-operatory HBS: high dose calcium + alphacalcidol
Hernandez 2021 [8]	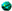 23-y female with RHPT 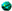 10-y history of peritoneal dialysis	Subtotal PTx → HBS (PTH ↘ to 205 pg/mL): high dose calcium + calcitriol
Lin 2020 [143]	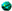 30-y female with RHPT	HBS (IV therapy of 17 days)
Bransky 2020 [136]	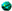 48-y female with RHPT and maxillary brown tumor (PTH = 4400 pg/mL)	TPT for severe hypocalcemia amid HBS
Ahmed 2020 [137]	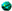 35-y female with RHPT and mandible brown tumor	TPT for persistent hypocalcemia amid HBS
Radu 2020 [31]	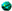 65-y male with RHPT 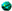 iPTH = 1257 pg/mL	HBS with severe hypocalcemia (2.2–3.1 mg/dL) with cardiac arrest
Wu 2019 [144]	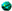 N = 10 p with RHPT + m-TOEPVA 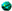 median age: 58.5 y	1/10 p with HBS (11-day hospitalization)
Tai 2019 [145]	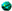 74-y male with RHPT 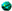 persistent post-operatory HPT due to ectopic PT adenoma	HBS after both interventions
Hassanein 2019 [25]	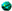 42-y female with RHPT 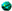 calciphylaxis as indication to perform PTx	HBS initially aggravated calciphylaxis which completely remitted in 1 y
Anwar 2018 [26]	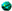 25-y female with RHPT 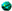 PTH = 1849 pg/mL 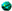 chronic anemia	HBS requiring extremely high doses of calcium (maximum of 35.9 g. day)
Tayyebi-Khosroshahi 2017 [146]	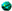 60-y male with RHPT 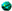 2 procedures of subtotal PTx	HBS following renal transplant after both PTx
Bashir 2016 [141]	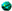 male adult with RHPT 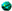 a14-y history of hemodialysis	calciphylaxis after therapy for HBS
Altun 2015 [147]	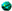 N = 3 p with RHPT and PTx + hemodialysis	Persistent HBS-related hypophosphatemia (8–10 months)
Crowley 2014 [121]	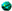 34-y female with tertiary HPT 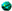 X-linked hypophosphataemia	HBS → normalization of renal phosphorus threshold
Hamrahian 2013 [111]	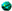 N = 2 p with tertiary HPT 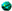 symmetrical craniofacial hypertrophy due to high doses of cinalcet (270 mg/day, respective 180 mg/day)	HBS
Park-Sigal 2013 [148]	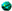 33-y female with tertiary PHT 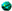 osteitis fibrosa cystica 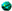 primary hyperaldosteronism	HBS
Chu 2011 [149]	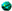 N = 3 p with PTx for RHPT and uremic tumoral calcinosis	HBS: 1 out of 3 patients
Goto 2010 [150]	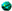 59-y female with RHPT	HBS (cinacalcet before PTx)
Collins 2005 [120]	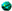 N = 3 p with mineral metabolism defects	HBS: 1 out of 3 patients (pseudohypoparathyroidism type 1b)
Ohlrich 2005 [151]	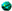 38-y female with RHPT and severe malnutrition	HBS
Savio 2004 [122]	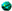 N = 6 p with X-linked dominant hypophosphatemic rickets	PTx: 3/6 and HBS
Miles 1997 [152]	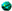 N = 1 with RHPT after 1 y since renal transplant	Prolonged HBS (20 months)
Hardoff 1996 [153]	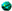 59-y male with RHPT and brown tumors	HBS
Frajewicki 1990 [154]	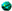 N = 1 p with RHPT	HBS with resistant hypophosphatemia → intraperitoneal phosphate therapy
Benz 1989 [155]	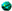 N = 3 p with RHPT	HBS with severe hypocalcemia (1 p with malabsorption) → intraperitoneal calcium therapy

Abbreviations: AP = alkaline phosphatase; Ca = calcium; HBS = hungry bone syndrome; HPT = hyperparathyroidism; iPTH = intact parathormone; N = number of patients; p = patient; PTx = parathyroidectomy; RHPT = renal hyperparathyroidism; TPT = teriparatide; TOEPVA = transoral endoscopic parathyroidectomy vestibular approach; y = years.

## Data Availability

Not applicable.

## Abbreviations

AP	alkaline phosphatase
ERAS	enhanced recovery after surgery
FGF-23	Fibroblast Growth Factor-23
m-TOEPVA	transoral endoscopic parathyroidectomy vestibular approach
PT	parathyroid
PTH	parathormone (parathyroid hormone)
PTx	parathyroidectomy
RHPT	renal hyperparathyroidism
PHPT	primary hyperparathyroidism
